# Relationship between serum uric acid levels and metabolism associated fatty liver disease in postmenopausal women based on NHANES 2017–2020

**DOI:** 10.1038/s41598-025-93738-3

**Published:** 2025-03-15

**Authors:** Xiaoding Zhou, Zongxiang Yue, Shuming He, Fengjuan Yuan, Xingrui He, Jiaqi Wang, Rong Wang, Ya Luo, Qiong Yi

**Affiliations:** 1 School of Clinical Medicine, Chengdu University of Traditional Chinese Medicine, Chengdu, 610075 China; 2 Center for Reproductive Medicine, Traditional Chinese Medicine Hospital of Meishan, Meishan, 620010 China

**Keywords:** Postmenopause, Fatty liver disease, Uric acid, Metabolism, NHANES, Metabolic disorders, Endocrinology, Endocrine system and metabolic diseases

## Abstract

Studies have shown that postmenopausal women have more metabolic abnormalities than premenopausal women. No consensus exists on how serum uric acid (sUA) affects metabolism-associated fatty liver disease (MAFLD) in postmenopausal women.This prospective observational study examined this link using National Health and Nutrition Examination Survey (NHANES) 2017 to 2020 data. We divided women’s sUA levels into four quartiles and used logistic regression, subgroup analyses, and restricted triple spline methods to compare the prevalence of MAFLD in postmenopausal and non-menopausal women. We also used histograms to analyze the effect of BMI-based indices. This population-based study involved 4477 women, including 1139 postmenopausal women aged 55–73 years. Multivariate logistic regression showed that, in the fully adjusted model, we found that participants in the highest quartile of sUA had a statistically significant 254% increased risk of MAFLD compared with participants in the lowest quartile (OR: 3.54; 95% CI 3.54 1.47–8.55; *P* < 0.001). Subgroup analyses showed no significant interaction between sUA levels and specific subgroups *P*( > 0.05 for all interactions). Additionally, RCS and threshold analysis showed a linear correlation (*P* = 0.186) and an ideal inflection point of 4.6 (*P* = 0.818) to the left. Right of the inflection point, the effect size was 1.524 (95% CI 1.291–1.814; *P* < 0.01). Histograms demonstrated that postmenopausal BMI increased sUA’s influence on MAFLD and higher sUA levels and BMI may enhance the prevalence of MAFLA in US postmenopausal women. The results of this study suggest that monitoring sUA levels in the postmenopausal period is critical in determining the occurrence of and interventions for MAFLD.

## Introduction

Non-alcoholic fatty liver disease (NAFLD) is dangerously common, reaching 30% worldwide^1^. NAFLD is a dangerous disorder that causes over 5% liver cell degeneration, but is not caused by alcohol consumption or viral infection. Over time, fat can accumulate in the liver, causing persistent damage^2^. A group of 31 experts from 22 nations advocated replacing NAFLD with MAFLD in 2020^3^, adding metabolic diseases to the diagnosis. MAFLD is anticipated to become the most common liver disease over the next decade as obesity rates rise and lifestyles change.

Nearly 1 billion women worldwide are postmenopausal, and menopause experiences vary greatly^4^. Hormonal changes after menopause cause physical and mental problems for many people. However, studies indicate that menopause induces changes in adipose tissue quantity and morphology, alongside alterations in blood lipid levels and insulin resistance^5^. After menopause, women experience hormonal changes, including reduced oestrogen levels and increased circulating androgens, as well as lifestyle modifications that lower basal metabolic rate compared to premenopausal stages^6^. Furthermore, the downregulation of estrogen receptors in visceral fat cells leads to increased androgen receptor expression, enhancing sensitivity to androgens and promoting visceral fat accumulation^7^. Excess visceral fat is associated with elevated lipolysis rates, producing free fatty acids that contribute to the development of insulin resistance^8^. Consequently, postmenopausal women are at increased risk of developing metabolic disorder-related diseases^9^. Evidence suggests that postmenopausal women have a higher likelihood of developing NAFLD compared to premenopausal women^10^. NAFLD is more common in men than premenopausal women under 50, according to studies. When women reach menopause, NAFLD incidence increases linearly, peaking at 60–69 years and declining after 70^11^. Meanwhile, postmenopausal women are 1.2 times more likely than males of the same age to develop NAFLD and progress to severe fibrosis^12^.

The sUA is a metabolite derived from purine oxidation, which occurs when its nucleotide is decomposed. Owing to the balance between the production and excretion of sUA, the concentration of sUA in serum remains relatively constant. Studies have shown that the increase in sUA content in the serum is related to the incidence of NAFLD^13^. Petta et al. reported that hyperuricemia can be used as an indicator to evaluate the severity of NAFLD - related liver injury^14^. A cross-sectional study in China indicated that in postmenopausal women with a normal BMI, an elevation in sUA levels, albeit being within the normal range, was positively associated with the occurrence of NAFLD^15^. The rising prevalence of MAFLD has imposed a significant strain on global healthcare systems. Given that MAFLD is a chronic condition requiring long-term therapy, additional study is essential to elucidate its associated pathogenic variables and mechanisms within the domain of postmenopausal-related disorders, which is crucial for advancing women’s health. The objective of this study was to assess the relationship between sUA levels and MAFLD in postmenopausal women by analyzing NHANES data from 2017 to March 2020, as well as to investigate additional influencing factors. It will also provide epidemiological data for continued subsequent investigation of the increased prevalence of MAFLD in postmenopausal women.

## Materials and methods

### Data source/study population

**Fig. 1:**
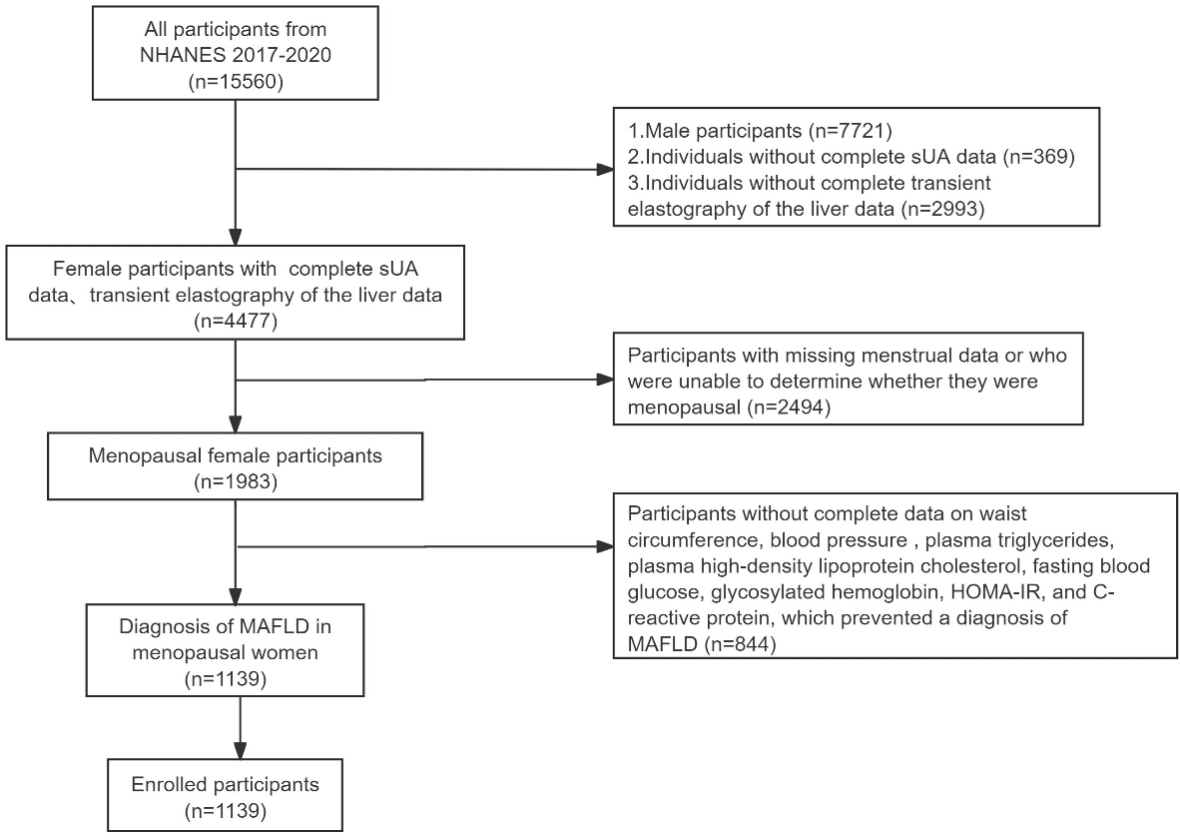
Flowchart of participant selection. NHANES National 2017–2020 MAFLD metabolism-associated fatty liver disease.

The study population used data from the NHANES (https://www.cdc.gov/nchs/nhanes/index.htm) from the U.S. Centers for Disease Control and Prevention, which included data from 2017 to 2020. Among the 15,560 subjects who participated in the study, we removed individuals of male sex (*n* = 7721), individuals without complete liver transient imaging data (*n* = 2494), and individuals without sUA content data (*n* = 369). Nonmenopausal women and individuals who did not have MAFLD (*n* = 844) were excluded. Finally, 1139 women participated in the study (Fig. 1).

### Variable

#### sUA levels

sUA, the final product of purines, is closely related to various health problems. In this study, sUA levels were divided into four quartiles (Q1: ≤4.2 mg/dL, Q2: 4.2 ~ 4.9 mg/dL, Q3: 5.0 ~ 5.9 mg/5.9 mg/dL, and Q:4 ≥ 5.9 mg/dL) to study the relationships between different sUA levels and the incidence of MAFLD. In this study, serum samples were collected from participants, stored at a temperature of -30 °C, and then transferred to the CDC, the NCEH, or the DLS for examination. The sUA levels were measured via the Beckman Coulter UniCel ^®^ DxC800 as part of a routine serum biochemical profile.

#### VCTE

Transient elastography of the liver (VCTE) is a widely used noninvasive and convenient method. The NHANES database uses the FibroScan 502 V2 Touch probe and equipment for examining liver ultrasound transient elastography. When the vibration point is in contact with the skin, it will generate shear waves through mechanical vibration. This wave travels faster through harder tissues and passes through the liver. Adipose tissue can lead to increased attenuation of ultrasound; therefore, by measuring the degree of attenuation of ultrasound (CAP), we can estimate fat content and indicate the degree of hepatic steatosis^16^. The subjects received VCTE. A medium (M) or large (XL) probe was used to collect 30 measurements for each participant. If (1) the individual fasted for at least 3 h before the test, (2) 10 or more complete liver stiffness measurements (LSMs) were performed, and (3) the quartile/median of the LSM was less than 30%, the test results were included in the dataset.

#### Diagnostic definition of MAFLD

The diagnosis of MAFLD was conducted on the basis of the 2020 consensus of the national expert panel. In brief, participants with hepatic steatosis who also presented any one of the following conditions were diagnosed with MAFLD: overweight/obesity, type 2 diabetes, or metabolic dysregulation. Metabolic dysregulation is defined by the presence of at least two metabolic risk abnormalities, including (1) white men and women with a waist circumference of ≥ 102/88 cm; (2) blood pressure ≥ 130/85 mmHg or specific medication treatment; (3) plasma triglycerides ≥ 150 mg/dL or specific drug treatment; (4) male plasma high-density lipoprotein cholesterol (HDL) < 40 mg/dL, female HDL < 50 mg/dL, or specific drug treatment; (5) prediabetes (fasting blood glucose 100 ~ 125 mg/dL or glycosylated hemoglobin 5.7% ~ 6.4%; (6) omeostatic model assessment of insulin resistance (HOMA-IR) ≥ 2.5; and (7) plasma high-sensitivity C-reactive protein (CRP) > 2 mg/L^17^. Although clinical guidelines recommend liver biopsy assessment as the gold standard for the diagnosis of liver disease, the number of MAFLD patients worldwide is currently large, and performing liver biopsy on a large number of people to assess liver disease status (such as hepatic steatosis) is unrealistic. In this study, to identify hepatic steatosis, a CAP of 248 dB/mm was selected as the critical threshold^18^. In addition, we collected liver stiffness measurements (LSMs) to assess liver fibrosis. The extent of fibrosis can be classified into three categories: F2, F3, and F4, with thresholds of 8.2, 9.7, and 13.6 kPa, respectively^16^.

#### Covariate

In this study, we selected covariates associated with MAFLD on the basis of previous studies. Demographic information is derived from the NHANES database, which contains data on age (in years), gender (classified as male or female), racial/ethnic background (including Mexican American, other Hispanic, white non-Hispanic, and black non-Hispanic), education level (classified as less than high school, high school or equivalent, some college or AA degree, and college graduate or above), marital status (classified as married/living with partner, widowed/divorced/separated, widowed/divorced/separated, never married), etc. This information was obtained from the NHANES demographic questionnaire. We obtained information on smoking status (yes/no) from the questionnaire. In addition, we collected glycosylated hemoglobin (HbA1c) (%), total cholesterol (TC) (mmol/L), triglyceride (TG) (mg/dL), systolic blood pressure (mmHg), diastolic blood pressure (mmHg), fasting blood glucose (mmol/L), fasting insulin (mmol/L), high-density lipoprotein (mmol/L), low-density lipoprotein (mmol/L), triglyceride (mg/dL), and C-reactive protein (mg/L) levels from laboratory examination data. In accordance with the criteria of the American Diabetes Association, participants were diagnosed with type 2 diabetes mellitus (T2DM) if they met any of the following conditions: (1) self-reported history of diagnosis; (2) use of antidiabetic drugs (including oral antidiabetic drugs or insulin); (3) HbA1c level ≥ 6.5%; (4) fasting blood glucose ≥ 7.0 mmol/L^19^; Criteria for hyperlipidemia according to the National Cholesterol Education Program-Adult Treatment Panel III, including low-density lipoprotein cholesterol (LDL-C) ≥ 130 mg/dL, TC ≥ 200 mg/dL, TG ≥ 150 mg/dL or high-density lipoprotein cholesterol (HDL-C) ≤ 50 mg/dL (female); Hypertension can be diagnosed when the subjects systolic blood pressure (SBP) ≥ 140 mmHg and/or diastolic blood pressure (DBP) ≥ 90 mmHg; self-reported history of hypertension; or self-reported current use of antihypertensive drugs^20^. The formula for the insulin resistance index was fasting insulin (uIU/ml) × fasting blood glucose (mmol/L)/22.5^21^. Finally, waist circumference (CM) and Body mass index (BMI) data were collected from body measurement data. BMI was used as an index of obesity in this study, and the participants were divided into two groups according to BMI (the nonobese group, with a BMI < 25 kg/m^2^, and the obese group, with a BMI ≥ 25 kg/m^2^) on the basis of the World Health Organization’s Asia–Pacific guidelines^19^. These variables are derived from the NHANES database for March 2017 to 2022.

#### Statistical analyses

Data were collected from the NHANES database from 2007 to 2020. Categorical variables are presented as frequencies and percentages, and continuous variables are presented as the means and standard deviations (SDs). Disparities in continuous and categorical variables were assessed via independent and chi-square tests, respectively. Multivariable logistic regression modeling were conducted to establish an association between sUA and MAFLD. Four models were developed: Model 1: unadjusted; Model 2: adjusted for age, smoking status, race, education, and marital status; Model 3: adjusted for age, smoking status, race, education, marital status, ALT, AST, and GGT; Model 4: adjusted for age, smoking status, race, education, marital status, alanine aminotransferase (ALT), aspartate aminotransferase (AST), gamma-glutamyltransferase (GGT), diabetes, hypertension, and hypercholesterolemia. Subgroup analyses were conducted to explore associations between sUA and MAFLD in different individuals. RCS was employed to visualize the potential linear or nonlinear relationship between sUA and MAFLD. In addition, the association between sUA and MAFLD was examined using histogram results derived from the study population’s characteristics, categorized by body mass index and menopausal status. The chi-square test was used to compare categorical variables between two groups, and the Cochran–Mantel–Henszel test was used to compare categorical variables among multiple groups. A Cochran‒Armitage trend test was used to compare the prevalence of MAFLD in subjects with different uric acid levels. In this study, the sUA data are presented according to quartiles.The software used to perform the analyses was R (version 4.2.3) and *P* < 0.05 was considered statistically significant.

## Result

### Baseline characteristics

A database from 2017 to 2020 in NHANES was utilized for this study, encompassing 15,560 potential participants. Additionally, 844 premenopausal women and non-MAFLD patients were excluded. This resulted in a total of 1,139 participants being included, with 633 patients being diagnosed with MAFLD (Fig. 1). Table 1 describes the demographic, socio-economic, physiological indicators of all female participants. The prevalence of MAFLD in the general population was 44.61%, with an average age of 44.77 ± 20.23 years, while the prevalence of MAFLD in postmenopausal women is 55.58%.

**Table 1:** Weighted baseline characteristics of all female participants.

Quartiles of sUA levels (mg/dL)	Overall	Q1	Q2	Q3	Q4	*p*-value
Number	4477	1222	1067	1131	1057	
Age (years)	44.77 (20.23)	39.10 (18.75)	42.79 (20.02)	45.37 (20.11)	52.69 (19.66)	*< 0.0001*
Wasit (cm)	96.71 (18.28)	88.30 (15.47)	93.28 (16.67)	99.27 (17.01)	107.53(18.27)	*< 0.0001*
BMI (kg/m^2^)	29.75 (8.27)	26.20 (6.43)	28.13 (7.10)	30.93 (8.12)	34.23 (9.05)	*< 0.0001*
Never smoking, n (%)	2668 (68.11)	730 (71.36)	648 (70.74)	671 (67.30)	619 (63.10)	*0.0002*
Marital status, n (%)						*< 0.0001*
Married/ Living with partner	1987 (52.96)	559 (58.11)	496 (57.01)	500 (51.98)	432 (45.09)	
Widowed/ Divorced/ Separated	1039 (27.69)	192 (19.96)	220 (25.29)	286 (29.73)	341 (35.59)	
Never married	726 (19.35)	211 (21.93)	154 (17.70)	176 (18.30)	185 (19.31)	
Alcohol use (%)						*0.409*
Never drinking (%)	142 (65.74)	32 (62.75)	30 (58.82)	44 (73.33)	36 (66.67)	
Heavy drinking	3 ( 1.39)	1 ( 1.96)	2 ( 3.92)	0 ( 0.00)	0 ( 0.00)	
Occasional drink	71 (32.87)	18 (35.29)	19 (37.25)	16 (26.67)	18 (33.33)	
Education, n (%)						*0.0512*
Less than high school	641 (17.08)	166 (17.24)	145 (16.63)	163 (16.96)	167 (17.43)	
High school or equivalent	865 (23.04)	215 (22.33)	190 (21.79)	226 (23.52)	234 (24.43)	
Some college or AA degree	1306 (34.79)	329 (34.16)	287 (32.91)	332 (34.55)	358 (37.37)	
College graduate or above	942 (25.09)	253 (26.27)	250 (28.67)	240 (24.97)	199 (20.77)	
Race/ ethnicity, n (%)						*< 0.0001*
Mexican American	583 (13.02)	204 (16.69)	137 (12.84)	137 (12.11)	105 ( 9.93)	
Other Hispanic	471 (10.52)	152 (12.44)	110 (10.31)	130 (11.49)	79 ( 7.47)	
White, non-Hispanic	1508 (33.68)	395 (32.32)	365 (34.21)	389 (34.39)	359 (33.96)	
Black, non-Hispanic	1157 (25.84)	296 (24.22)	248 (23.24)	285 (25.20)	328 (31.03)	
Other race	758 (16.93)	175 (14.32)	207 (19.40)	190 (16.80)	186 (17.60)	
Other factor						
SBP (mmHg)	119.55(20.18)	114.79(18.21)	118.04(18.90)	120.05(19.85)	126.03(22.15)	*< 0.0001*
DBP (mmHg)	72.50 (11.32)	69.87 (10.33)	71.64 (11.07)	73.62 (11.12)	75.23 (12.10)	*< 0.0001*
FBG (mmol/L)	2.95 (3.29)	2.67 (3.32)	2.87 (3.20)	3.03 (3.11)	3.30 (3.47)	*0.0001*
HLD (mmol/L)	1.48 (0.41)	1.55 (0.40)	1.52 (0.41)	1.46 (0.41)	1.39 (0.40)	*< 0.0001*
HbA1c (%)	5.73 (1.00)	5.62 (1.14)	5.63 (0.93)	5.70 (0.80)	5.99 (1.07)	*< 0.0001*
Triglyceride (mg/dL)	95.95 (62.65)	79.24 (48.95)	86.79 (51.26)	101.00(72.12)	115.97(67.58)	*< 0.0001*
AST (U/L)	19.64 (11.55)	18.90 (10.35)	18.72 (10.47)	19.71 (10.38)	21.34 (14.53)	*< 0.0001*
ALT (U/L)	17.61 (13.98)	15.99 (11.91)	16.46 (16.05)	18.10 (12.76)	20.13 (14.81)	*< 0.0001*
GGT (IU/L)	24.29 (34.75)	20.53 (33.32)	20.73 (22.11)	24.21 (30.15)	32.31 (47.70)	*< 0.0001*
HOME-IR	2.10 (6.02)	1.37 (3.20)	1.84 (5.81)	2.19 (5.75)	3.12 (8.41)	*< 0.0001*
CRP (mg/L)	4.26 (8.25)	2.83 (5.34)	3.60 (9.65)	4.66 (7.18)	6.13 (9.99)	*< 0.0001*
Diabetes, n (%)	706(15.77)	124(10.15)	126(11.81)	178(15.74)	278(26.3)	*< 0.0001*
Hypertension, n (%)	1657 (37.01)	276(22.59)	328 (30.74)	421(37.22)	632 (59.79)	*< 0.0001*
Hyperlipidemia, n (%)	3007 (67.17)	673 (55.07)	672 (62.98)	804 (71.09)	858 (81.17)	*< 0.0001*
MAFLD, n (%)	1997 (44.61)	337 (27.58)	392 (36.74)	580 (51.28)	688 (65.09)	*< 0.0001*
Liver fibrosis, n (%)						*0.6248*
F2, n (%)	123 (34.26)	24 (43.64)	18 (36.00)	29 (35.80)	52 (30.06)	
F3, n (%)	134 (37.33)	16 (29.09)	20 (40.00)	29 (35.80)	69 (39.88)	
F4, n (%)	102 (28.41)	15 (27.27)	12 (24.00)	23 (28.40)	52 (30.06)	

Q1: ≤4.2 mg/dL; Q2: 4.2~4.9 mg/dL,; Q3: 5.0~5.9 mg/5.9 mg/dL; Q:4≥5.9 mg/dL. Mean ± SD for continuous variables: P value was calculated by weighted ANOVA test. % for categorical variables: P value was calculated by weighted chi-square test. Median [interquartile range] for continuous variables: P value was calculated by weighted Kruskal-Wallis H test. BMI, Body mass index; SBP, Systolic blood pressure; DBP, Diastolic blood pressure; FBG, Fasting blood glucose; HLD, High-density lipoprotein cholesterol; HbA1c, Glycosylated hemoglobin ; AST, Aspartate aminotransferase; ALT, Alanine aminotransferase; GGT, Gamma-glutamyltransferase; HOME-IR, Omeostatic model assessment of insulin resistance; CRP, Plasma high-sensitivity C-reactive protein; F2, F3, and F4, with thresholds of 8.2, 9.7, and 13.6 kPa, respectively.

Table 2 shows the baseline characteristics of postmenopausal women. Subjects with higher sUA levels were more likely to have a higher prevalence of MAFLD (Q1: 43.13%, Q2: 50.19%, Q3: 60.51%, Q4: 69.61%). However, the increase of sUA concentration and the probability of liver fibrosis did not show significant statistical significance. Otherwise, the prevalence of diabetes and hypertension is higher. (*P* < 0.001).Compared with premenopausal women, postmenopausal women have higher levels of SBP, DBP, FBG, HLD, triglyceride, AST, ALT, GGT, IR-HOME, CRP, and increase with the increase of sUA level (*P* < 0.001), which means that the abnormal rate of metabolic related indicators in postmenopausal women is higher.

**Table 2:** Weighted baseline characteristics of postmenopausal women.

Quartiles of sUA levels (mg/dL)	Overall	Q1	Q2	Q3	Q4	*p*-value
Number	1139	313	267	276	283	
age	64.30 (9.04)	63.27 (8.52)	64.13 (9.44)	64.58 (9.09)	65.31 (9.06)	*0.0452*
Wasit (cm)	100.56(16.03)	94.07 (13.58)	98.39 (14.55)	102.22(14.14)	108.41(18.03)	*< 0.0001*
BMI (kg/m^2^)	30.13 (7.38)	27.34 (5.93)	29.19 (6.20)	30.61 (6.65)	33.62 (8.92)	*< 0.0001*
Never smoking (%)	708 (62.16)	205 (65.50)	176 (65.92)	174 (63.04)	153 (54.06)	*0.0114*
Marital status (%)						*0.0019*
Married/ Living with Partner	572 (50.35)	170 (54.66)	140 (52.63)	148 (53.62)	114 (40.28)	
Widowed/ Divorced/ Separated	459 (40.40)	121 (38.91)	105 (39.47)	104 (37.68)	129 (45.58)	
Never married	105 ( 9.24)	20 ( 6.43)	21 ( 7.89)	24 ( 8.70)	40 (14.13)	
Education (%)						*0.0697*
Less than high school	214 (18.82)	46 (14.74)	53 (19.85)	60 (21.82)	55 (19.43)	
High school or equivalent	287 (25.24)	76 (24.36)	78 (29.21)	67 (24.36)	66 (23.32)	
Some college or AA degree	342 (30.08)	91 (29.17)	70 (26.22)	83 (30.18)	98 (34.63)	
College graduate or above	294 (25.86)	99 (31.73)	66 (24.72)	65 (23.64)	64 (22.61)	
Race/ ethnicity, n (%)						*0.0006*
Mexican American	115 (10.10)	34 (10.86)	33 (12.36)	31 (11.23)	17 ( 6.01)	
Other Hispanic	133 (11.68)	43 (13.74)	38 (14.23)	28 (10.14)	24 ( 8.48)	
White, non-Hispanic	446 (39.16)	132 (42.17)	109 (40.82)	103 (37.32)	102 (36.04)	
Black, non-Hispanic	268 (23.53)	62 (19.81)	44 (16.48)	68 (24.64)	94 (33.22)	
Other race	177 (15.54)	42 (13.42)	43 (16.10)	46 (16.67)	46 (16.25)	
Other factor						
SBP (mmHg)	131.22(20.74)	128.89(20.08)	131.48(20.49)	131.85(20.51)	133.00(21.77)	*0.1158*
DBP (mmHg)	74.86 (10.82)	73.76 (10.69)	74.92 (10.29)	75.57 (10.11)	75.37 (11.98)	*0.1926*
FBG (mmol/L)	3.13 (3.47)	3.13 (3.82)	2.86 (3.04)	3.08 (3.23)	3.44 (3.65)	*0.2688*
HLD (mmol/L)	1.54 (0.43)	1.64 (0.45)	1.59 (0.44)	1.50 (0.40)	1.42 (0.40)	*< 0.0001*
HbA1c (%)	6.07 (1.10)	6.10 (1.41)	5.90 (0.77)	5.94 (0.74)	6.33 (1.21)	*< 0.0001*
Triglyceride (mg/dL)	110.67(57.50)	104.30(59.72)	102.97(51.99)	117.01(60.61)	117.95(55.83)	*0.0436*
AST (U/L)	21.54 (10.75)	20.94 (8.40)	22.31 (11.40)	21.35 (9.74)	21.67 (13.11)	*0.4786*
ALT (U/L)	19.72 (13.54)	18.22 (9.79)	20.29 (14.09)	20.18 (15.08)	20.38 (14.89)	*0.1497*
GGT (IU/L)	29.60 (41.77)	25.16 (35.78)	26.79 (32.06)	30.19 (37.76)	36.58 (56.44)	*0.0052*
HOME-IR	2.13 (5.94)	1.59 (3.30)	1.64 (2.73)	2.32 (8.90)	3.02 (6.67)	*0.0107*
CRP (mg/L)	4.70 (10.90)	4.04 (14.74)	4.41 (8.59)	4.76 (8.37)	5.63 (10.02)	*0.336*
Diabetes (%)	289 (25.37)	73 (23.32)	48 (17.98)	64 (23.19)	104 (36.75)	*< 0.0001*
Hypertension (%)	718 (63.04)	64 (51.12)	152 (56.93)	177 (64.13)	229 (80.92)	*< 0.0001*
Hyperlipidemia, n (%)	963 (84.55)	249 (79.55)	224 (83.90)	236 (85.51)	254 (89.75)	*0.007*
MAFLD, n (%)	633 (55.58)	135 (43.13)	134 (50.19)	167 (60.51)	197 (69.61)	*< 0.0001*
Liver fibrosis, n (%)						*0.2977*
F2, n (%)	42 (33.33)	11 (52.38)	6 (31.58)	9 (29.03)	16 (29.09)	
F3, n (%)	48 (38.10)	6 (28.57)	5 (26.32)	15 (48.39)	22 (40.00)	
F4, n (%)	36 (28.57)	4 (19.05)	8 (42.11)	7 (22.58)	17 (30.91)	

Q1: ≤4.2 mg/dL; Q2: 4.2~4.9 mg/dL,; Q3: 5.0~5.9 mg/5.9 mg/dL; Q:4≥5.9 mg/dL. Mean ± SD for continuous variables: P value was calculated by weighted ANOVA test. % for categorical variables: P value was calculated by weighted chi-square test. Median [interquartile range] for continuous variables: P value was calculated by weighted Kruskal-Wallis H test. BMI, Body mass index; SBP, Systolic blood pressure; DBP, Diastolic blood pressure; FBG, Fasting blood glucose; HLD, High-density lipoprotein cholesterol; HbA1c, Glycosylated hemoglobin ; AST, Aspartate aminotransferase; ALT, Alanine aminotransferase; GGT, Gamma-glutamyltransferase; HOME-IR, Omeostatic model assessment of insulin resistance; CRP, Plasma high-sensitivity C-reactive protein; F2, F3, and F4, with thresholds of 8.2, 9.7, and 13.6 kPa, respectively.

### Logistic regression analysis of the association between sUA and MAFLD

Table 3 presents the ORs and 95% CIs for the relationship between sUA levels and MAFLD in postmenopausal women following logistic regression analysis. According to the unadjusted model, a significant correlation of 3.15 (1.98 ~ 5.03) was observed between sUA and MAFLD. Specifically, for each 1-unit increase in sUA, there was a 215% increase in the risk of MAFLD compared with baseline. In the multivariate regression model, after adjusting for various factors, such as age, smoking status, race, education, marital status, ALT, AST, GGT, diabetes, hypertension and hypercholesterolemia, the ORs ranged from 1.46 to 4.77, indicating a strong correlation between sUA levels and MAFLD, with consistent results (*p* < 0.001). Sensitivity analysis was performed with sUA quartiles, and the OR for Q1, Q2, Q3, and Q4 in Model 4 was 3.54 (1.47 ~ 8.55). Compared with those in Quartile 1, participants in Quartile 4 were associated with a 254% increased risk of MAFLD (p for trend < 0.01).

**Table 3:** Relationship between sUA and MAFLD in postmenopausal women.

	Model 1	Model 2	Model 3	Model 4
	OR (95%CI)	OR (95% CI)	OR (95% CI)	OR (95% CI)
sUA level	3.15 (1.98 ~ 5.03)***	3.25(1.93 ~ 5.45)***	3.20 (1.87 ~ 5.48)***	2.64 (1.46 ~ 4.77)**
Quartiles				
Quartiles1	Ref	Ref	Ref	Ref
Quartiles2	1.98 (1.21 ~ 3.25)**	1.87 (1.09 ~ 3.22)*	1.87 (1.07 ~ 3.27)*	1.92 (1.02 ~ 3.57)*
Quartiles3	3.47 (1.98 ~ 6.09)***	3.30 (1.80 ~ 6.13)**	3.20 (1.80 ~ 5.91)**	3.12 (1.52 ~ 6.35)**
Quartiles4	4.66 (2.53 ~ 8.58)***	4.81 (2.35 ~ 9.88)***	4.70 (2.20 ~ 10.17)**	3.54 (1.47 ~ 8.55)*
P for trend	*P* < 0.001	*P* < 0.001	*P* < 0.001	*P* < 0.001

Model 1: unadjusted; Model2: adjusted by age, smoking, race, education, marital status; Model 3: adjusted by age, smoking, race, education, marital status, ALT, AST, GGT; Model4: adjusted by age, smoking, race, education, marital status, ALT, AST, GGT, diabetes, hypertension, hypercholesterolemia. *P < 0.05; **P < 0.01; ***P < 0.001.

We also performed logistic regression analysis between sUA levels and MAFLD in premenopausal women (Table 4). In premenopausal individuals, sUA levels were also positively correlated with the occurrence of MAFLD. According to the univariate analysis, all four models revealed that premenopausal uric acid levels had a smaller effect on MAFLD than did postmenopausal women. In the multivariate regression analysis, Model 3 and Model 4 also yielded the same results. In addition, according to the sensitivity analysis performed with sUA quartiles, the effect of an increase in sUA level on MAFLD in premenopausal women was not as significant as that in postmenopausal women. The incidence of MAFLD in postmenopausal women with high sUA levels was 147% greater than that in premenopausal women .

**Table 4:** Relationships between sUA levels and the MAFLD in premenopausal women.

	Model 1	Model 2	Model 3	Model 4
	OR (95% CI)	OR (95%CI)	OR (95% CI)	OR (95% CI)
sUA level	3.07 (2.59 ~ 3.64)***	2.82 (2.21 ~ 3.61)***	1.95 (1.44 ~ 2.64)***	1.75 (1.25 ~ 2.45)**
Quartiles				
Quartiles1	Ref	Ref	Ref	Ref
Quartiles2	1.71 (1.36 ~ 2.15)**	1.68 (1.29 ~ 2.18)**	1.18 (0.88 ~ 1.57)	1.13 (0.82 ~ 1.56)
Quartiles3	3.60 (2.70 ~ 4.82)**	3.54 (2.36 ~ 5.30)***	2.28 (1.48 ~ 3.51)**	2.16 (1.34 ~ 3.50)**
Quartiles4	5.68 (4.47 ~ 7.22)**	4.77 (3.38 ~ 6.72)***	2.26 (1.78 ~ 3.96)**	2.07 (1.32 ~ 3.26)**
P for trend	*P* < 0.001	*P* < 0.001	*P* < 0.001	*P* < 0.001

Model 1: unadjusted; Model 2: adjusted by age, smoking, race, education, marital status; Model 3: adjusted by age, smoking, race, education, marital status, ALT, AST, GGT; Model 4: adjusted by age, smoking, race, education, marital status, ALT, AST, GGT, diabetes, hypertension, hypercholesterolemia. P* < 0.05; **P < 0.01; ***P < 0.001.

### Subgroup analysis

**Fig. 2:**
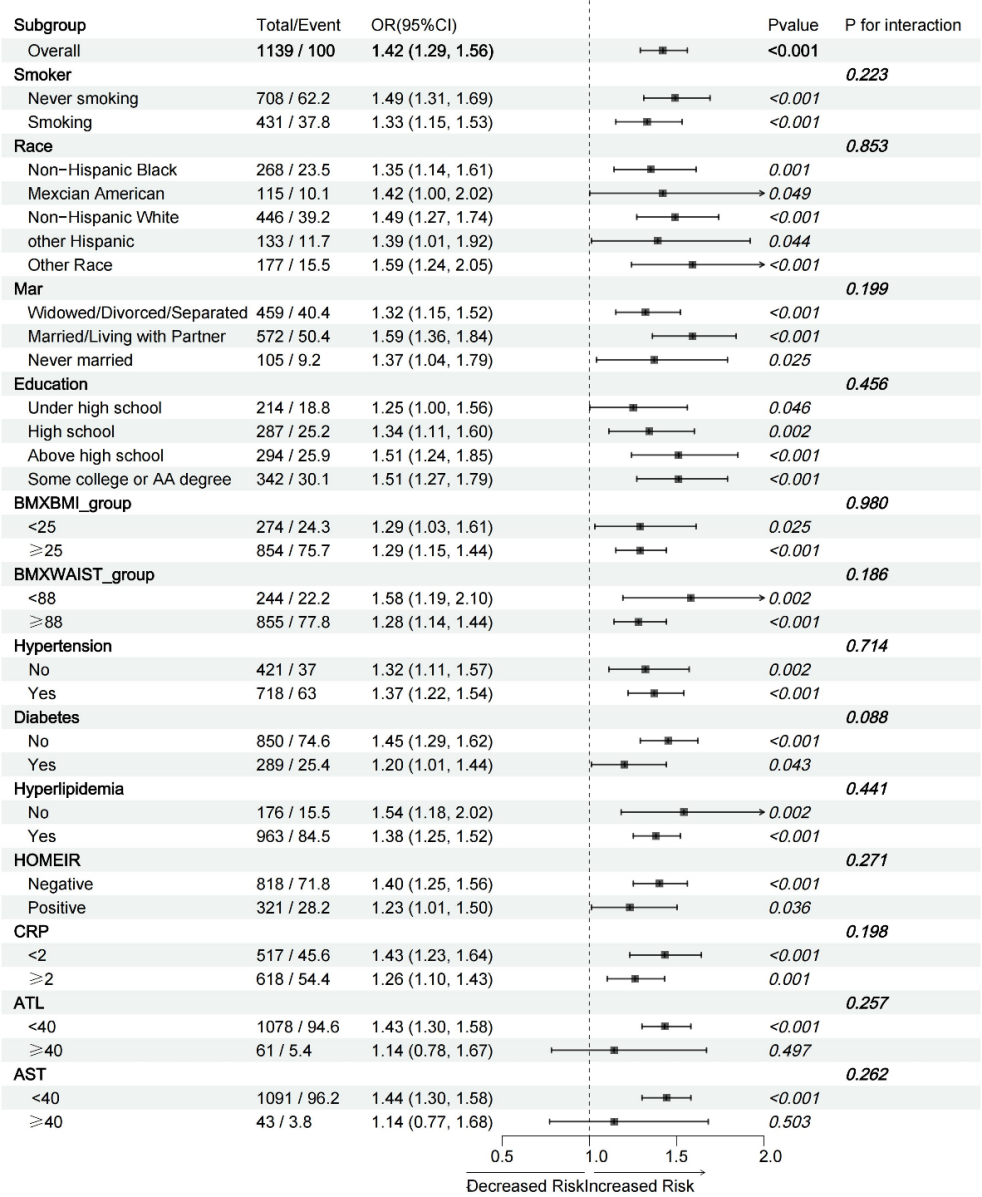
Subgroups analyses. In the subgroup analysis stratified by race, education level, smoking status, marital status, BMI, waist circumference, hypertension, diabetes, hyperlipidaemia, insulin index, CRP, ATL, ASL, and ASL/ATL, the model is not adjusted forrace, education level, smoking status, marital status, BMI, waist circumfeence, hypertension, diabetes, hyperlipidaemia, insulin index, CRP, ATL and ASL respectively.

We conducted subgroup analyses to evaluate the consistency of the relationship between sUA levels and MAFLD prevalence across different groups. Interaction tests revealed no significant interactions between sUA and MAFLD across the subgroups (all *P* > 0.05) (Fig. 2). This indicates that factors such as race (Mexican American/White, non-Hispanic/Black, non-Hispanic/Other Hispanic/Other race), education level (Less than high school/High school or equivalent/Some college or AA degree/College graduate or above), smoking status (Never smoking), marital status (Married/Living with partner/Widowed/ Divorced/ Separated/Never married), body mass index (< 25, ≥ 25), waist circumference (< 88, ≥ 88), hypertension (yes/no), diabetes (yes/no), hyperlipidemia (yes/no), insulin index (yes/no), CRP (< 2, ≥ 2), ALT(< 40, ≥ 40), and AST (< 40, ≥ 40) did not significantly influence the association between sUA and MAFLD risk. The consistent association observed across all subgroups underscores the stability and reliability of the findings, further supporting the conclusion that sUA is an independent risk factor for developing MAFLD.

#### The nonlinear correlation between the MAFLD and the level of sUA

**Fig. 3:**
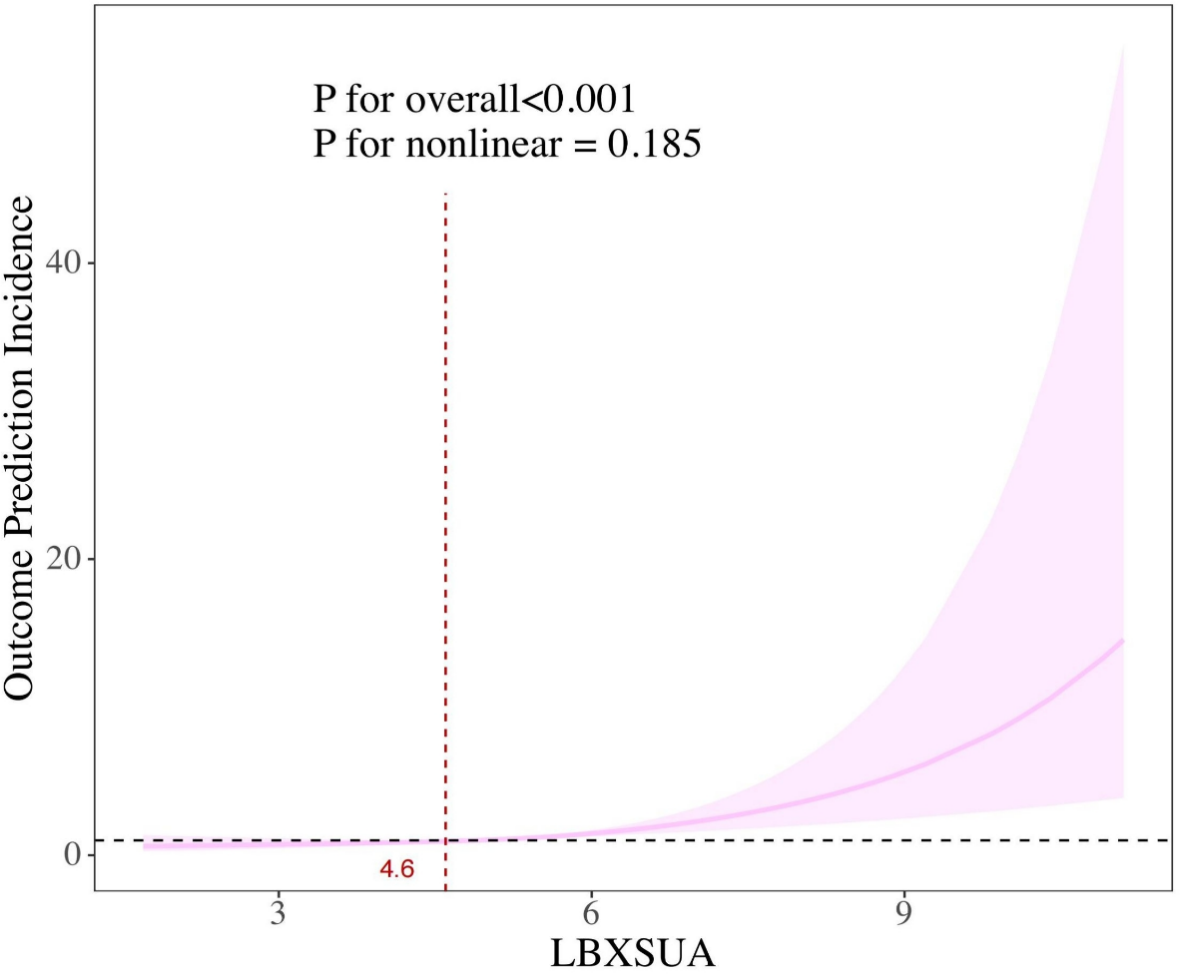
The smooth curve fitting between the prevalence of MAFLD and sUA levels. The x-axis represents sUA.The y-axis represents the 95% confidence interval from the fit. The dashed line indicates an OR of 1, which represents no association between prevalence of MAFLD and sUA levels. The model adjusted by age, race, education, marital, smoking, BMI, waistline.

The findings indicated a linear correlation between the incidence of MAFLD and sUA levels (*P for nonlinear = 0.186*) (Fig. 3). Table 5 presents the effect thresholds derived from both the standard linear regression model and the two-piecewise linear regression model, indicating that the optimal inflection point between the MAFLD occurrence and sUA levels is 4.6. To the left of the inflection point P was 0.818, indicating a lack of statistically significant relationship between the two variables. To the right of the inflection point, the effect size was 1.524 (1.291–1.814) (*P* < 0.01), suggesting that elevated sUA levels were significantly correlated with an increased risk of MAFLD. A 1-unit increase in sUA was linked to a 52.4% rise in the risk of MAFLD.

**Table 5:** Threshold effect analysis of the MAFLD and sUA levels.

Outcome	The effect size, 95% CI	*P* value
Model 1 Fitting model by standard linear regression	1.369 (1.221–1.539)	*< 0.01*
Model 2 Fitting model by two-piecewise linear regression		
Inflection point	4.6	
< 4.6	1.039 (0.747–1.441)	*0.818*
> 4.6	1.524 (1.291–1.814)	*< 0.01*
*P* for likelihood ratio test	0.077	
Model 1 Fitting model by standard linear regression	1.369 (1.221–1.539)	*< 0.01*

#### Relationships between the occurrence of MAFLD and the level of sUA according to BMI and menopausal status

**Fig. 4:**
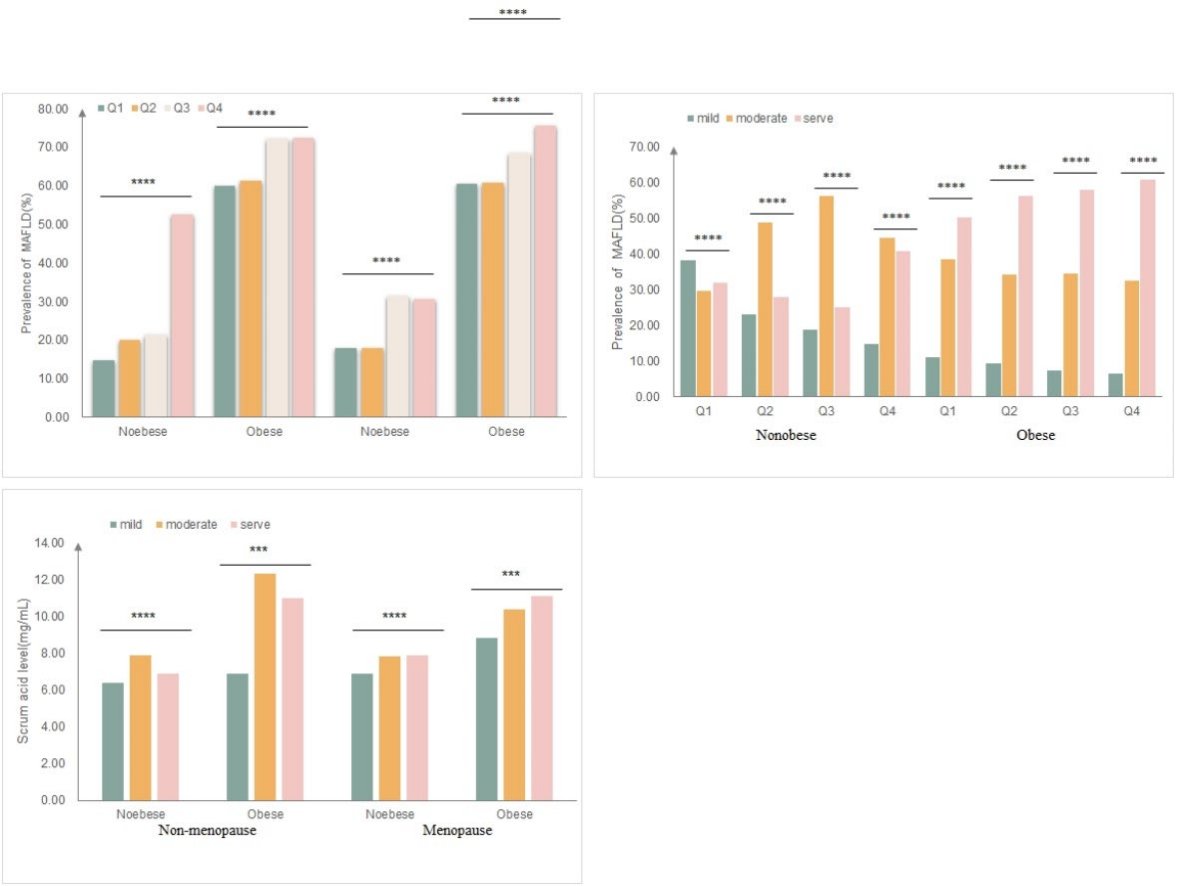
(**A**) Prevalence of MAFLD according to sUA level quartiles in postmenopausal women; (**B**) The prevalence of different grades of MAFLD according to sUA level quartiles in postmenopausal women; (**C**) sUA levels of postmenopausal women patients according to grades of MAFLD; Q1:≤4.2 mg/dL; Q2: 4.2 ~ 4.9 mg/dL; Q3:5.0 ~ 5.9 mg/dL; Q4: ≥5.9 mg/dL. *P < 0.05; **P < 0.01; ***P < 0.001.

We assessed the occurrence of MAFLD in both nonobese and obese individuals, distinguishing between nonmenopausal and postmenopausal groups (Fig. 4A). In the postmenopausal cohort, a nonobese patient exhibited no significant positive correlation between increased sUA levels and the incidence of MAFLD. This contradicts the findings of earlier research, potentially linked to the sample size characteristics in this study. The incidence of MAFLD in both the postmenopausal and nonmenopausal groups did not show a significant positive correlation with uric acid levels attributable to weight .

Subjects with MAFLD were categorized into three groups based on abdominal ultrasonography results: mild (248–301 dB/mL), moderate (302–307 dB/mL), and severe (> 307 dB/mL). Figure 4B presents a stratified analysis of BMI concerning the incidence of MAFLD across varying severities, categorized by quartiles of sUA in postmenopausal women. Obese individuals exhibited a more pronounced increase in sUA and a higher incidence of severe MAFLD. In the sUA content group, obesity exacerbated the severity of MAFLD.

Furthermore, the stratified analysis of BMI revealed no significant positive correlation between sUA levels and the severity of MAFLD in premenopausal women. In both nonobese and obese postmenopausal women, a significant positive correlation and dose-response relationship exist between sUA levels and the severity of liver disease in patients with MAFLD (*P* < 0.0001) (Fig. 4C).

## Discussion

A cross-sectional analysis utilizing NHANES data from 2017 to March 2020 demonstrated a significant correlation between sUA levels and the prevalence of MAFLD among menopausal women in the United States. Elevated sUA levels were consistently linked to higher rates of MAFLD in various menopausal populations. This finding has significant clinical implications, indicating that sUA may serve as a risk factor for MAFLD. Monitoring sUA levels in postmenopausal women enables clinicians to detect individuals at high risk for developing MAFLD early, facilitating timely interventions to mitigate this risk. These findings validate and deepen the hypotheses initially proposed in this study, highlighting the complex interactions between sUAs levels and the development of MAFLD in postmenopausal women and thus provide important insights into the reduction of metabolism-related diseases in postmenopausal women and the management of postmenopausal women’s health.

In recent studies, the terms NAFLD and MAFLD are often used interchangeably. However, according to international definitions, MAFLD places greater emphasis on the concept of metabolic dysfunction compared to NAFLD^22^. A retrospective study suggests that menopausal status may contribute to the onset and progression of NAFLD^23^. Several mechanisms have been proposed by prior studies to explain how menopausal status could be linked to an increased risk of NAFLD. Sex hormones, particularly estrogen, are steroid hormones that bind to hepatocyte receptors. In women of childbearing age, estrogen plays a protective role against excessive hepatic fat accumulation by stimulating lipolysis and inhibiting hepatic lipogenesis^24^. Estrogen also promotes the excretion of excess free fatty acids by facilitating the removal of oxidized very-low-density lipoproteins and stimulates the synthesis of HDL, thereby maintaining hepatic cholesterol homeostasis^25^. Additionally, estrogen enhances mitochondrial function, promotes the oxidation of free fatty acids, improves insulin sensitivity, supports glycolipid metabolic processes, and reduces intrahepatic inflammation^26^. Animal studies have shown that estrogen-deficient mice are more susceptible to lipid metabolism abnormalities, which lead to hepatic fat accumulation^27^. In our study, cross-sectional data analysis revealed that postmenopausal women exhibited a higher prevalence of MAFLD compared to premenopausal women, consistent with previous findings.

Over the past few years, several emerging studies have examined the relationship between MAFLD and sUA levels. These studies suggest that the risk of NAFLD in women with different menstrual statuses increases as sUA levels rise^28^. Previous research has established a positive association between elevated sUA levels and the development of NAFLD, though most of these studies have focused on a broader range of age groups and included both men and women, with limited application of the concept of MAFLD^29,30^. Furthermore, there is a paucity of studies that specifically address the relationship between MAFLD and sUA in postmenopausal women using publicly available databases. In contrast, our study, based on multivariate logistic regression analyses, RCS, and threshold analyses, demonstrates a significant positive association between serum uric acid levels and the risk of MAFLD in postmenopausal women in the United States. Potential mechanisms through which elevated sUA levels are positively associated with MAFLD in postmenopausal women include several factors. Studies have shown that estrogen levels influence the urate transport system in the kidneys, with increased estrogen leading to enhanced sUA levels clearance through increased secretion and inhibition of reabsorption^31^. However, perimenopausal and postmenopausal women tend to exhibit higher sUA levels^32^. The pathways through which sUA contributes to the development of MAFLD are complex and involve insulin resistance, oxidative stress, and inflammatory responses. High sUA levels impair insulin signaling through inhibition of intrahepatic IRS1 and Akt pathways, thereby inducing insulin resistance. This, in turn, leads to an increase in cytotoxic substances and lipid peroxidation products in hepatocytes, promoting hepatic fat accumulation^33,34^. Additionally, sUA exacerbates mitochondrial oxidative stress, inhibits activity in the tricarboxylic acid cycle, and induces fat deposition in hepatocytes^35^. Moreover, sUA stimulates the production of reactive oxygen species by activating NADPH oxidases, particularly NOX4, leading to aberrant activation of NLRP3, which contributes to hepatic steatosis and inflammation^36^. Previous studies have also demonstrated that sUA and cytosolic NOX trigger a cascade of endoplasmic reticulum stress, which promotes the release of the adipogenic transcription factor SREBP-1c, a regulator of adipogenic enzyme expression and lipid metabolism disorders^37^. This suggests that elevated sUA levels may serve as a useful biomarker for identifying patients with MAFLD and hepatic steatosis or related metabolic abnormalities. In our study, the highest prevalence of MAFLD (69.61%) was observed in the Q4 subgroup (quartile with the highest sUA levels) of postmenopausal women, which may be attributed to the decreased estrogen levels leading to higher sUA levels. Furthermore, the threshold analysis revealed that the optimal inflection point for the relationship between MAFLD prevalence and sUA levels was 4.6 mg/dL. To the left of this inflection point, no statistically significant association was found (*p* = 0.818). However, beyond this threshold, a positive correlation emerged, with each 1-unit increase in sUA associated with a 52.4% increase in the risk of MAFLD. Therefore, sUA levels may serve as a valuable marker for assessing MAFLD risk in postmenopausal women.

Our study revealed that higher BMI was associated with elevated sUA levels and a greater prevalence of severe MAFLD in postmenopausal women, whereas no significant correlation was observed in premenopausal women. Previous studies have demonstrated that adipose tissue distribution, particularly abdominal fat, is strongly linked to hepatic steatosis and metabolic syndrome^38^. Postmenopausal women are more susceptible to weight gain, fat redistribution, and dyslipidemia, potentially due to hormonal changes^39^. Interestingly, our findings indicated no significant positive correlation in the postmenopausal cohort, which contrasts with results from prior studies. This discrepancy may stem from the characteristics of the sample in our database. While numerous studies suggest that BMI or sUA levels can be risk factors for MAFLD, evidence linking these variables specifically to menopausal women remains scarce, and the mechanisms underlying the interaction between obesity, sUA, and MAFLD are not well understood. A previous mediator analysis indicated that sUA accounted for 10% of obesity-associated NAFLD development, suggesting that elevated sUA may serve as a primary pathophysiological mechanism in the increased prevalence of NAFLD among obese adults^40^. This association may be linked to insulin resistance (IR), which disrupts the balance of renal urate reabsorption and excretion via stimulation of urate-anion exchange. Additionally, elevated sUA amplifies the pro-inflammatory response of adipose cells, heightening the risk of metabolic disorders^41^. This study provides a preliminary exploration of the relationship among these variables by BMI stratification, illustrated via a bar chart, which suggests that in postmenopausal women, higher BMI intensifies the impact of sUA on MAFLD development. For postmenopausal women with elevated sUA levels, weight loss could potentially reduce the incidence of MAFLD; however, further large-scale studies are necessary to validate this hypothesis.

This study has several limitations that require attention. The discussion regarding the influence of pertinent metabolism-related markers on MAFLD is insufficient in our study, making it impossible to ascertain whether other metabolic abnormalities affect uric acid levels and subsequently worsen the progression of MAFLD. The cross-sectional design of the study utilizing NHANES data limited our ability to establish causality, highlighting the necessity for future prospective cohort studies to corroborate our findings.

## Conclusion

This study demonstrated a significant positive correlation between sUA levels and MAFLD in postmenopausal women, providing compelling evidence that sUA may serve as a key indicator for assessing MAFLD risk in this population. Moreover, weight management in postmenopausal women could mitigate the risk of MAFLD, even at similar sUA levels. However, the cross-sectional design of this study limits the ability to infer causality. Future research should focus on developing diagnostic algorithms based on sUA data and validating these algorithms by comparing predicted outcomes with clinical diagnoses using the same datasets. Despite its limitations, this study underscores the potential utility of sUA in MAFLD risk assessment and highlights the need for diverse, global research to build on these findings.

## Data Availability

Publicly available datasets were analyzed in this study. This data can be found here: https://www.cdc.gov/nchs/nhanes/index.htm.

## References

[1] Younossi, Z. M. et al. The global epidemiology of nonalcoholic fatty liver disease (NAFLD) and nonalcoholic steatohepatitis (NASH): A systematic review. *Hepatology* **77**, 1335–1347. 10.1097/HEP.0000000000000004 (2023). 36626630 10.1097/HEP.0000000000000004 PMC10026948

[2] Rinella, M. E. et al. AASLD practice guidance on the clinical assessment and management of nonalcoholic fatty liver disease. *Hepatology* **77**, 1797–1835. 10.1097/HEP.0000000000000323 (2023). 36727674 10.1097/HEP.0000000000000323 PMC10735173

[3] Eslam, M. et al. MAFLD: A consensus-driven proposed nomenclature for metabolic associated fatty liver disease. *Gastroenterology* **158**, 1999–2014. 10.1053/j.gastro.2019.11.312 (2020). 32044314 10.1053/j.gastro.2019.11.312

[4] Brown, L. et al. Promoting good mental health over the menopause transition. *Lancet* **403**, 969–983. 10.1016/S0140-6736(23)02801-5 (2024). 38458216 10.1016/S0140-6736(23)02801-5

[5] Khalfa, A. et al. Prevalence of metabolic syndrome and its association with lifestyle and cardiovascular biomarkers among postmenopausal women in Western Algeria. *Int. J. Gynaecol. Obstet.* **138** (2), 201–206. 10.1002/ijgo.12206 (2017). 28494104 10.1002/ijgo.12206

[6] Ko, S. H. & Jung, Y. Energy metabolism changes and dysregulated lipid metabolism in postmenopausal women. *Nutrients* **13** (12), 4556. 10.3390/nu13124556 (2021). 34960109 10.3390/nu13124556 PMC8704126

[7] Jeong, H. G. & Park, H. Metabolic disorders in menopause. *Metabolites* **12** (10), 954. 10.3390/metabo12100954 (2022). 36295856 10.3390/metabo12100954 PMC9606939

[8] Stefanska, A., Bergmann, K. & Sypniewska, G. Metabolic syndrome and menopause: Pathophysiology, clinical and diagnostic significance. *Adv. Clin. Chem.* **72**, 1–75. 10.1016/bs.acc.2015.07.001 (2015). 26471080 10.1016/bs.acc.2015.07.001

[9] Danpanichkul, P. et al. Metabolic syndrome and metabolic Dysfunction-Associated steatotic liver disease in premenopausal women: Global trends and projections to 2040. *Mayo Clin. Proc.* **99** (10), 1615–1628. 10.1016/j.mayocp.2023.12.025 (2024). 38551541 10.1016/j.mayocp.2023.12.025

[10] Polyzos, S. A. et al. Menopause and metabolic dysfunction-associated steatotic liver disease. *Maturitas* 108024 10.1016/j.maturitas.2024.108024 (2024). 10.1016/j.maturitas.2024.108024 38760254

[11] Younossi, Z. M. et al. Global epidemiology of nonalcoholic fatty liver disease—meta-analytic assessment of prevalence, incidence, and outcomes. *Hepatology* **64**, 73–84. 10.1002/hep.28431 (2016). 26707365 10.1002/hep.28431

[12] Balakrishnan, M. et al. Women have a lower risk of nonalcoholic fatty liver disease but a higher risk of progression vs men: A systematic review and meta-analysis. *Clin. Gastroenterol. Hepatol.* **19**, 61–71. 10.1016/j.cgh.2020.04.067 (2021). 32360810 10.1016/j.cgh.2020.04.067 PMC8796200

[13] Xu, C. et al. High serum uric acid increases the risk for nonalcoholic fatty liver disease: A prospective observational study. *PLoS ONE*. **5**, e11578. 10.1371/journal.pone.0011578 (2010). 20644649 10.1371/journal.pone.0011578 PMC2904389

[14] Petta, S. et al. Hyperuricemia is associated with histological liver damage in patients with non-alcoholic fatty liver disease. *Aliment. Pharmacol. Ther.* **34**, 757–766. 10.1111/j.1365-2036.2011.04788.x (2011). 21790685 10.1111/j.1365-2036.2011.04788.x

[15] Liu, P. J. et al. Relationship between serum uric acid levels and hepatic steatosis in non-obese postmenopausal women. *Climacteric* **17**, 692–699. 10.3109/13697137.2014.926323 (2014). 24884478 10.3109/13697137.2014.926323

[16] Barr, R. G. et al. Elastography Assessment of liver fibrosis: Society of radiologists in ultrasound consensus conference statement. *Radiology* **276**, 845–861 10.1148/radiol.2015150619 (2015). 10.1148/radiol.2015150619 26079489

[17] Eslam, M. et al. A new definition for metabolic dysfunction-associated fatty liver disease: An international expert consensus statement. *J. Hepatol.* **73**, 202–209. 10.1016/j.jhep.2020.03.039 (2020). 32278004 10.1016/j.jhep.2020.03.039

[18] Karlas, T. et al. Individual patient data meta-analysis of controlled Attenuation parameter (CAP) technology for assessing steatosis. *J. Hepatol.* **66**, 1022–1030. 10.1016/j.jhep.2016.12.022 (2017). 28039099 10.1016/j.jhep.2016.12.022

[19] American Diabetes Association. 2. Classification and diagnosis of diabetes: Standards of medical care in Diabetes-2020. *Diabetes Care* **43**, S14–S31 10.2337/dc20-S002 (2020). 10.2337/dc20-S002 31862745

[20] Kasanagottu et al. Predictors of treatment intensification in uncontrolled hypertension. *J. Hypertens.* **42**, 283–291. 10.1001/jama.2020.14545 (2024). 37889569 10.1097/HJH.0000000000003598

[21] Matthews, D. R. et al. Homeostasis model assessment: Insulin resistance and β-cell function from fasting plasma glucose and insulin concentrations in man. *Diabetologia* **28**, 412–419. 10.1007/BF00280883 (1985). 3899825 10.1007/BF00280883

[22] Lin, S. U. et al. Comparison of MAFLD and NAFLD diagnostic criteria in real world. *Liver Int.* **40** (9), 2082–2089 (2020). 32478487 10.1111/liv.14548

[23] Eng, P. C. et al. Non-alcoholic fatty liver disease in women - Current knowledge and emerging concepts. *JHEP Rep.* **5** (10), 100835. 10.1016/j.jhepr.2023.100835 (2023). 37771547 10.1016/j.jhepr.2023.100835 PMC10522907

[24] Chen, K. L. Madak-Erdogan. Estrogens and female liver health. *Steroids* **133**, 38–43. 10.1016/j.steroids.2017.10.015 (2018). 29100781 10.1016/j.steroids.2017.10.015

[25] Yang, M., Ma, F. & Guan, M. Role of steroid hormones in the pathogenesis of nonalcoholic fatty liver disease. *Metabolites* **11**, 320. 10.3390/metabo11050320 (2021). 34067649 10.3390/metabo11050320 PMC8156407

[26] Della Torre, S. Beyond the X factor: Relevance of sex hormones in NAFLD pathophysiology. *Cells* **10**(9), 2502 10.3390/cells10092502 (2021). 10.3390/cells10092502 PMC8470830 34572151

[27] Hart-Unger, S. et al. Hormone signaling and fatty liver in females: Analysis of Estrogen receptor α mutant mice. *Int. J. Obes.* **41**, 945–954. 10.1038/ijo.2017.50 (2017). 10.1038/ijo.2017.50 PMC5735425 28220039

[28] Chen, Y. et al. Association between serum uric acid and non-alcoholic fatty liver disease according to different menstrual status groups. *Can. J. Gastroenterol. Hepatol.* 2763093. 10.1155/2019/2763093 (2019). 10.1155/2019/2763093 PMC6906828 31871925

[29] Bao, T. et al. Association between serum uric acid and nonalcoholic fatty liver disease in Nonobese postmenopausal women: A cross-sectional study. *Sci. Rep.* **10**(1), 10072 10.1038/s41598-020-66931-9 (2020). 32572126 10.1038/s41598-020-66931-9 PMC7308322

[30] Yang, H. et al. Joint associations of serum uric acid and ALT with NAFLD in elderly men and women: A Chinese cross-sectional study. *J. Transl. Med.* **16**, 1–10. 10.1186/s12967-018-1657-6 (2018). 30333032 10.1186/s12967-018-1657-6 PMC6192201

[31] Li, R. et al. Uric acid metabolic disorders in Pituitary-Target gland axis. Diabetes. *Metaboli. Syndrome Obes.* **7**, 661–673. https://orcid.org/0000-0002-9864-444X (2024). 10.2147/DMSO.S448547 PMC10859102 38343584

[32] Wang, Renwei, et al. "Association between serum uric acid and bone mineral density in males from NHANES 2011–2020." *Scientific Reports* **14**(1), 4292. https://orcid.org/0000-0002-9864-444X (2024). 10.1038/s41598-024-52147-8 PMC10881460 38383617

[33] Evans, J. L., Maddux, B. A. & Goldfine, I. D. The molecular basis for oxidative stress-induced insulin resistance. *Antioxid. Redox. Signal.* **7** (7–8), 1040–1052. 10.1089/ars.2005.7.1040 (2005). 15998259 10.1089/ars.2005.7.1040

[34] Vacca, M., Allison, M., Griffin, J. L. & Vidal-Puig A fatty acid and glucose sensors in hepatic lipid metabolism: Implications in NAFLD. *Semin. Liver Dis.* **35**, 250–261. 10.1055/s-0035-1562945 (2015). 26378642 10.1055/s-0035-1562945

[35] Wang, Z., Xu, M., Hu, Z. & Shrestha, U. K. Prevalence of nonalcoholic fatty liver disease and its metabolic risk factors in women of different ages and body mass index. *Menopause* **22**, 667–673. 10.1097/GME.0000000000000352 (2015). 25513983 10.1097/GME.0000000000000352

[36] Tschopp, J. & Schroder, K. NLRP3 inflammasome activation: The convergence of multiple signalling pathways on ROS production?. *Nat. Rev. Immunol.* **10** (3), 210–215. 10.1038/nri2725 (2010). 20168318 10.1038/nri2725

[37] Choi, Y. J. et al. Uric acid induces fat accumulation via generation of Endoplasmic reticulum stress and SREBP-1c activation in hepatocytes. *Lab. Invest.* **94** (10), 1114–1125. 10.1038/labinvest.2014.98 (2014). 25111690 10.1038/labinvest.2014.98

[38] Chen, K. L. & Madak-Erdogan. Estrogens and female liver health. *Steroids* **133**, 38. 10.1016/j.steroids.2017.10.015 (2018). 29100781 10.1016/j.steroids.2017.10.015

[39] Grygiel-Górniak, B. et al. Uric acid and obesity-related phenotypes in postmenopausal women. *Mol. Cell. Biochem.* **443**, 111–119. 10.1007/s11010-017-3215-6 (2018). 29075989 10.1007/s11010-017-3215-6 PMC5943388

[40] Zhang, Q. et al. Serum uric acid is a mediator of the association between obesity and incident nonalcoholic fatty liver disease: A prospective cohort study. *Front. Endocrinol.* **12**, 657856. 10.3389/fendo.2021.657856 (2021). 10.3389/fendo.2021.657856 PMC8158156 34054728

[41] Yuan, H. Serum uric acid levels and risk of metabolic syndrome: A dose-response meta-analysis of prospective studies. *J. Clin. Endocrinol. Metabol.*. **100** (11), 4198–4207. 10.1210/jc.2015-2527 (2015). 10.1210/jc.2015-2527 26308292

